# *In vivo* Quantification of the Structural Changes of Collagens in a Melanoma Microenvironment with Second and Third Harmonic Generation Microscopy

**DOI:** 10.1038/srep08879

**Published:** 2015-03-09

**Authors:** Pei-Chun Wu, Tsung-Yuan Hsieh, Zen-Uong Tsai, Tzu-Ming Liu

**Affiliations:** 1 Institute of Biomedical Engineering, National Taiwan University, Taipei 10617, Taiwan; 2 Molecular Imaging Center, National Taiwan University, Taipei 10617, Taiwan

## Abstract

Using *in vivo* second harmonic generation (SHG) and third harmonic generation (THG) microscopies, we tracked the course of collagen remodeling over time in the same melanoma microenvironment within an individual mouse. The corresponding structural and morphological changes were quantitatively analyzed without labeling using an orientation index (OI), the gray level co-occurrence matrix (GLCM) method, and the intensity ratio of THG to SHG (*R*_THG/SHG_). In the early stage of melanoma development, we found that collagen fibers adjacent to a melanoma have increased OI values and SHG intensities. In the late stages, these collagen networks have more directionality and less homogeneity. The corresponding GLCM traces showed oscillation features and the sum of squared fluctuation *Var_GLCM_* increased with the tumor sizes. In addition, the THG intensities of the extracellular matrices increased, indicating an enhanced optical inhomogeneity. Multiplying OI, *Var_GLCM_,* and *R*_THG/SHG_ together, the combinational collagen remodeling (CR) index at 4 weeks post melanoma implantation showed a 400-times higher value than normal ones. These results validate that our quantitative indices of SHG and THG microscopies are sensitive enough to diagnose the collagen remodeling *in vivo*. We believe these indices have the potential to help the diagnosis of skin cancers in clinical practice.

Because of the heterogeneity of tumors, it is difficult to eradicate them using cytotoxic therapy alone^1^. Surviving clones can proliferate and result in further metastases after treatment^2^,^3^,^4^,^5^,^6^,^7^,^8^,^9^,^10^,^11^,^12^,^13^. In contrast to cytotoxic therapy, cytostatic therapy focuses on restraining tumor progression and lethal metastasis through a modification of the tumor microenvironment^14^. The cytostatic therapies targeted on critical molecules were specific to the pathophysiology of the developing cancer cells^15^,^16^. The tumor microenvironment is essentially composed of tumor cells and the surrounding non-tumor cells, including fibroblasts, endothelial cells, immune cells, and connective tissues, as well as the extracellular matrix (ECM)^17^. In the case of metastatic tumors, cells that migrate toward vessels or lymph nodes must cross the barriers of collagen networks. The structures of the collagen environment are thus remodeled in the process of cancer invasion and metastasis^18^,^19^,^20^. Collagen remodeling is primarily associated with the action of matrix metalloproteinases (MMPs), the main function of which is to decompose the protein components of the ECM, such as proteoglycans, glycoprotein, and collagen. However, it has been reported that carcinoma cells can secrete MMP-resistant homotrimers (α1/α1/α1 chains) to facilitate the migration of these cancer cells^9^,^21^. Reports have also shown that normal curly collagen fibers (heterotrimers) become thicker and linear (homotrimers) after tumor invasion^9^,^22^,^23^,^24^,^25^,^26^. These findings regarding the morphological changes of tumor collagens may potentially assist in the diagnosis of fatal metastases.

The traditional methods for the analysis of collagen remodeling, such as the weight measurement and colorimetric methods^27^,^28^, may destroy collagen structures and lead to diagnostic errors. The bright-field microscope can *ex vivo* analyze the collagen structures on fixed, collagen labeled, and thin-sliced ECM. With the advances in ultra-fast optics, second harmonic generation (SHG) and third harmonic generation (THG) microscopy provide non-invasive ways to analyze ECM remodeling *in vivo* or *in vitro* without using exogenous labels^29^,^30^,^31^,^32^,^33^. The SHG and THG microscopy have the advantages of decreased photodamage, superior optical penetration, and the ability to provide quantitative information^19^,^34^,^35^,^36^,^37^,^38^. However, most of the previous studies did not monitor collagen remodeling in the same tumor, and none of these studies use THG contrast to analyze collagen remodeling. Those studies routinely sacrificed animals or obtained biopsies at different stages of tumor growth^29^,^39^. Individual, regional, or depth variations might thus hide the cancer related changes of collagen structures. To time-course track exactly the same tumor microenvironment, investigators usually adopted an invasive window chamber setup to overcome the opaque skin and took sectioning images to observe collagen remodeling *in vivo*^19^,^30^,^31^,^32^,^33^. However, a bio-incompatible window chamber may result in an inflammatory environment, and the contact with the rigid glass may affect the collagen patterns through mechanotransduction^40^,^41^. Very few reports have simultaneously conducted non-invasive and time-course tracking of collagen remodeling in the same tumor microenvironment.

In this study, we used least-invasive harmonic generation microscopy (HGM) to continuously observe collagen remodeling induced by melanoma *in vivo*. We located melanoma cells *in vivo* through their intrinsic two-photon fluorescence (TPF) and THG contrasts. In the melanoma microenvironment, the distribution of collagen was revealed by the SHG microscopy. Elastin fibers^42^ and the optical inhomogeneity of the ECM were revealed by the THG microscopy. From these sectioning images, we used the following three different methods (See Methods section) to quantitatively analyze the collagen remodeling *in vivo*: the orientation index (OI)^34^,^43^,^44^, the gray level co-occurrence matrix (GLCM)^35^,^45^,^46^,^47^,^48^, and the ratio of the THG to the SHG (*R*_THG/SHG_). We found these indices have significant changes over baseline variations, which together can potentially provide an effective diagnosis of the developmental stages of melanoma-associated collagens.

## Results

**Figure 1:**
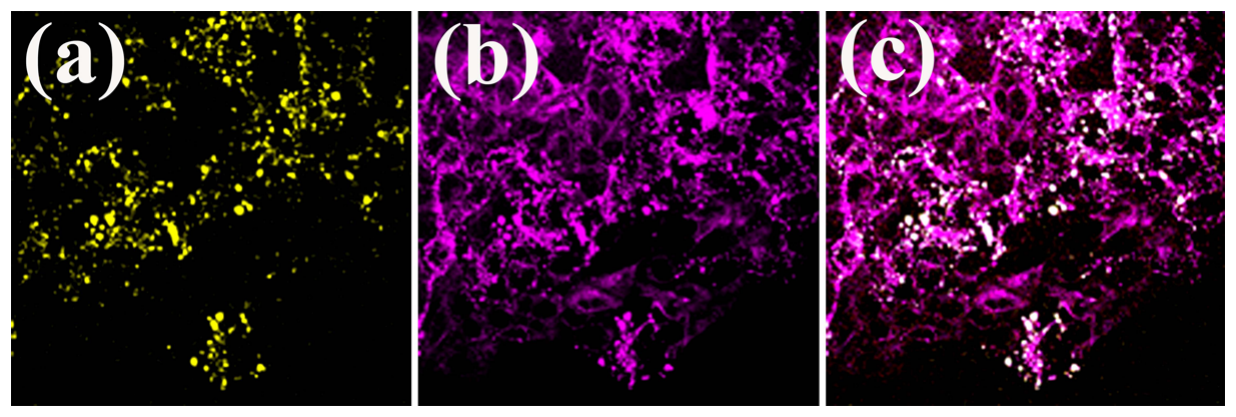
*In vitro* (a) TPF, (b) THG, and (c) combined images of melanoma cells.

**Figure 2:**
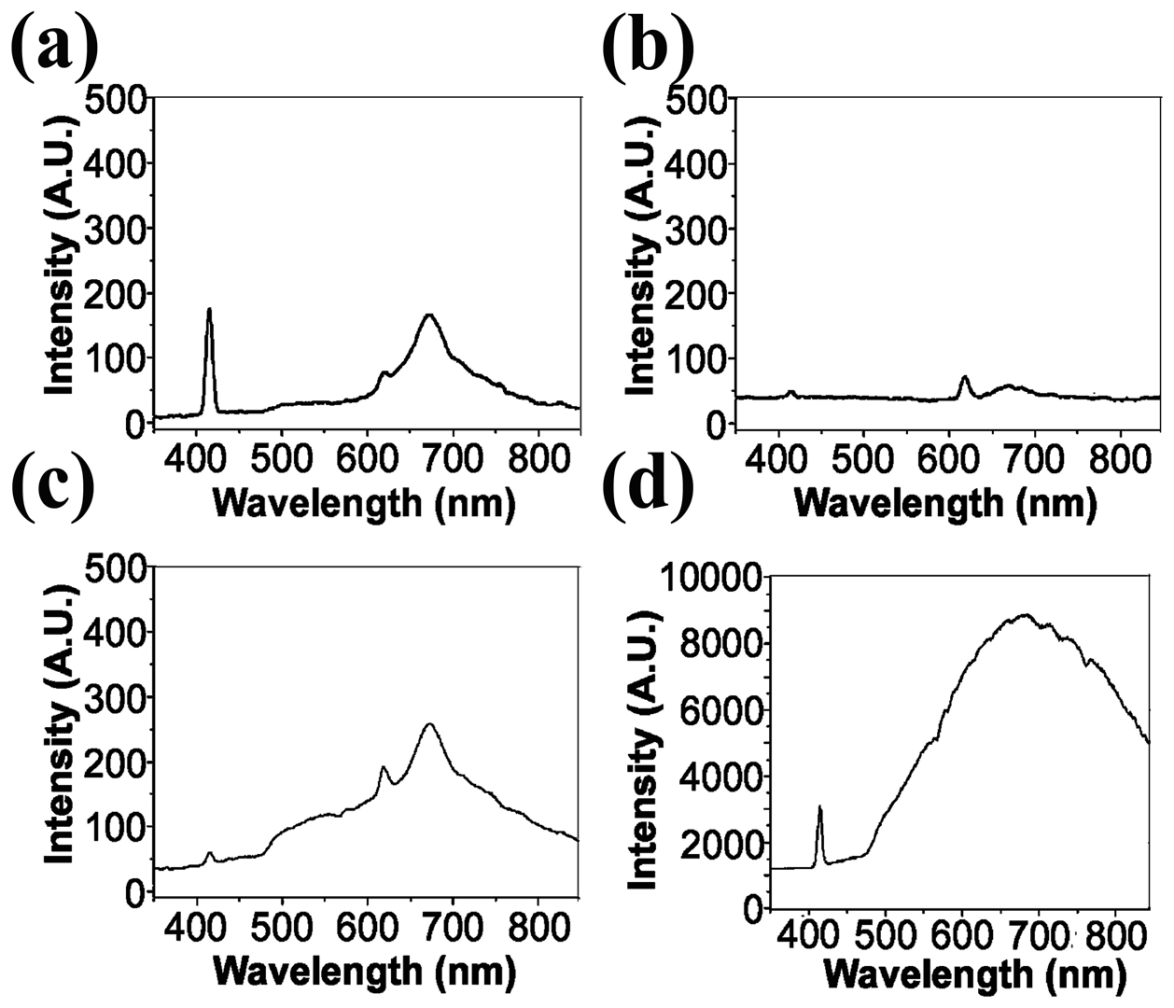
Two-photon fluorescence spectra of (a) melanoma cells, (b) medium before culture, (c) medium after culture, and (d) pure melanin. The narrow peaks around 417 nm and 625 nm are the THG of the glass-solution interfaces and the SHG of aggregated materials on glass, respectively.

In order to locate melanoma cells *in vivo* and analyze surrounding microenvironment, we need a fluorescence contrast specific to melanoma cells. Using the nonlinear optical microscopy to observe, the melanoma cells revealed an intrinsic THG^49^ and TPF contrasts with a granular appearance (Fig. 1). The TPF spectrum of the granules peaked at approximately 680 nm (Fig. 2a). This granular red autofluorescence was much weaker in the absence of melanoma cells (Fig. 2b, serum only). Collecting the culture media right above the melanoma cells, the secreted granules also had the same TPF spectrum (Fig. 2c). Both of these melanoma-related TPF spectra had peak wavelengths close to that of commercial melanin (Fig. 2d). These results suggest the granules might be melanosomes encapsulating melanin. In the following experiments, we exploited this characteristic TPF of melanin to track the melanoma cells *in vivo*.

### *In vivo* imaging collagen structures in normal mice ears

**Figure 3:**
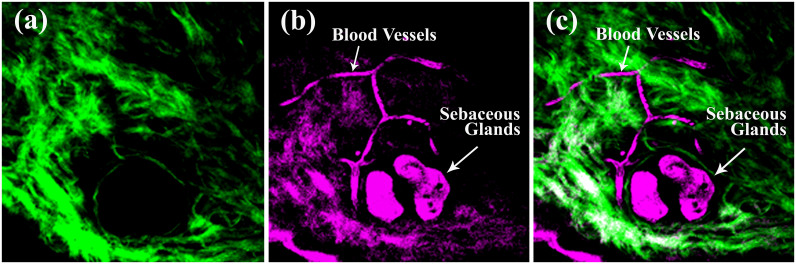
*In vivo* (a) SHG, (b) THG, and (c) combined imaging of a normal mouse ear. Fields of view: 240 μm × 240 μm.

Our nonlinear optical microscopy system can perform *in vivo* sectioning images of mouse skin without a label^50^,^51^,^52^. The imaging depth in skin tissues can be as deep as 350 μm. In general, SHG microscopy reveals structural proteins like collagen fibers (Fig. 3a). The intensity is dependent on the density of the collagen, the orientation of the fibers, and the laser polarization. In contrast, THG contrasts are sensitive to optical inhomogeneities^16^,^53^. They have been used to reveal adipocytes^51^, immune cells^52^, keratinocytes, basal cells^49^, elastin fibers^54^, and red blood cells (RBCs)^55^. The modality of THG is also sensitive to lipids^56^. An ultra-strong THG contrast can be found with sebaceous glands due to the high lipid content of these tissues (Fig. 3b). In blood vessels, the SHG signal is absent, and we can observe circulating RBCs with the THG contrast (Fig. 3).

**Figure 4:**
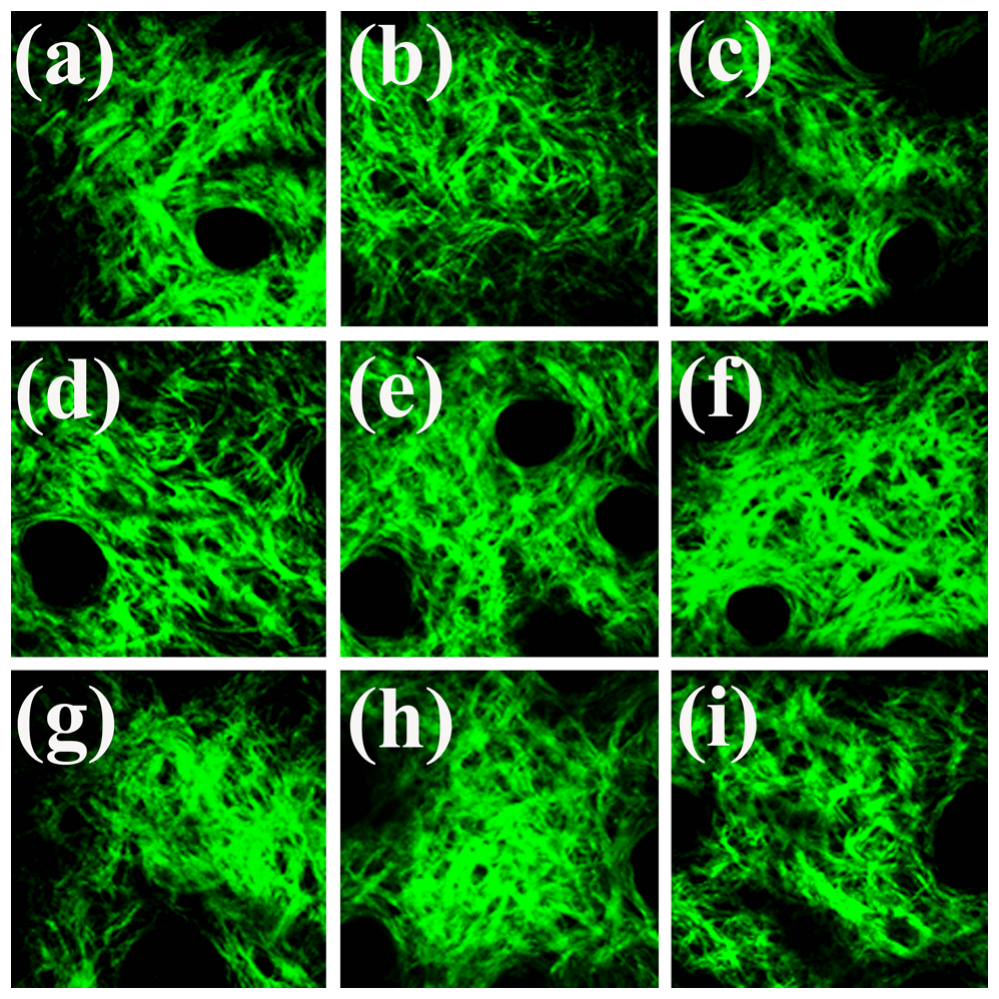
*In vivo* SHG imaging of the collagen networks in a normal mouse ear at (a) zone 1, (b) zone 2, (c) zone 3, (d) zone 4, (e) zone 5, (f) zone 6, (g) zone 7, (h) zone 8, and (i) zone 9. Fields of view: 240 μm × 240 μm. These images were acquired for control analysis.

Before implanting the melanoma cells in the mice ears, we obtained SHG sectioning images of the collagen fibers *in vivo* at nine different zones as a normal control (Fig. 4). The way we define the zones is described in the supplementary information. In each zone, we arbitrarily chose three locations for tomographic imaging. At each location, ten sectioning images at different depths were acquired.

### Observation of collagen remodeling within the same ear region

**Figure 5:**
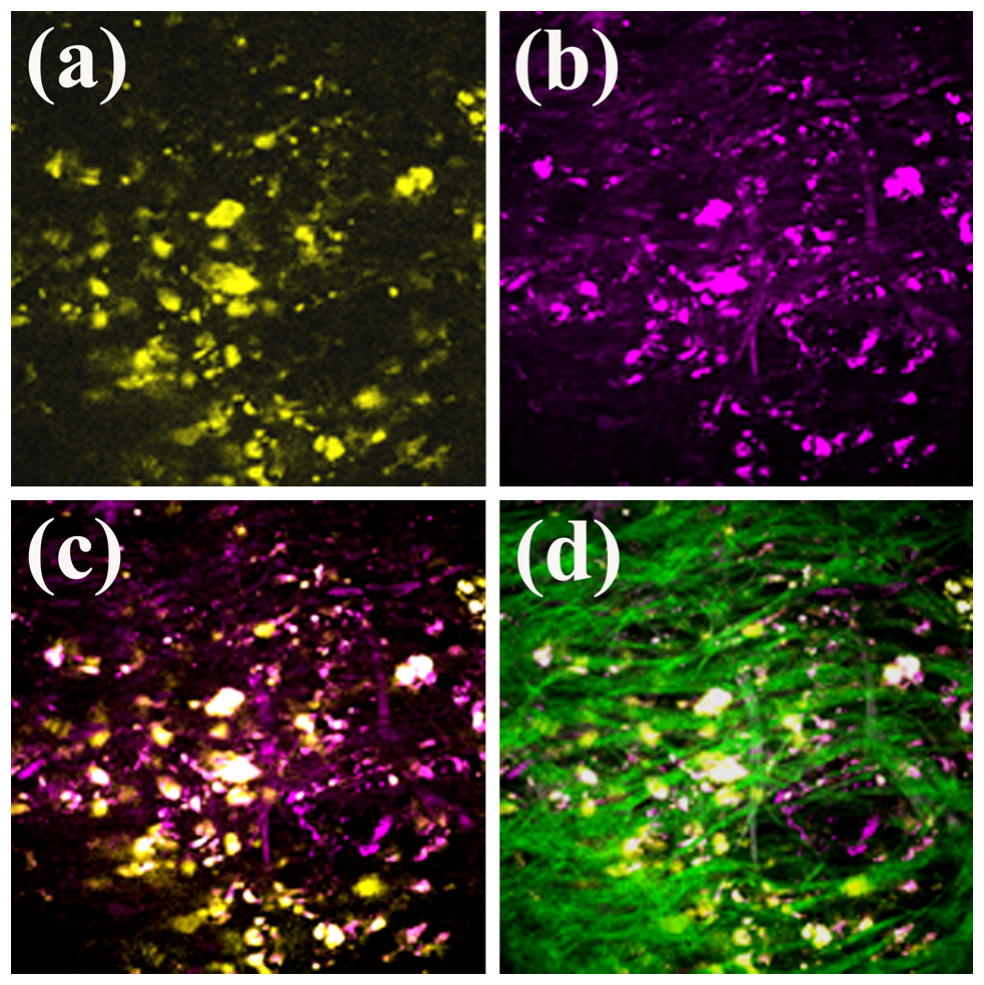
*In vivo* (a) TPF, (b) THG, (c) combined TPF and THG, and (d) combined TPF, THG, and SHG (green) imaging of melanoma cells in the mouse ear.

**Figure 6:**
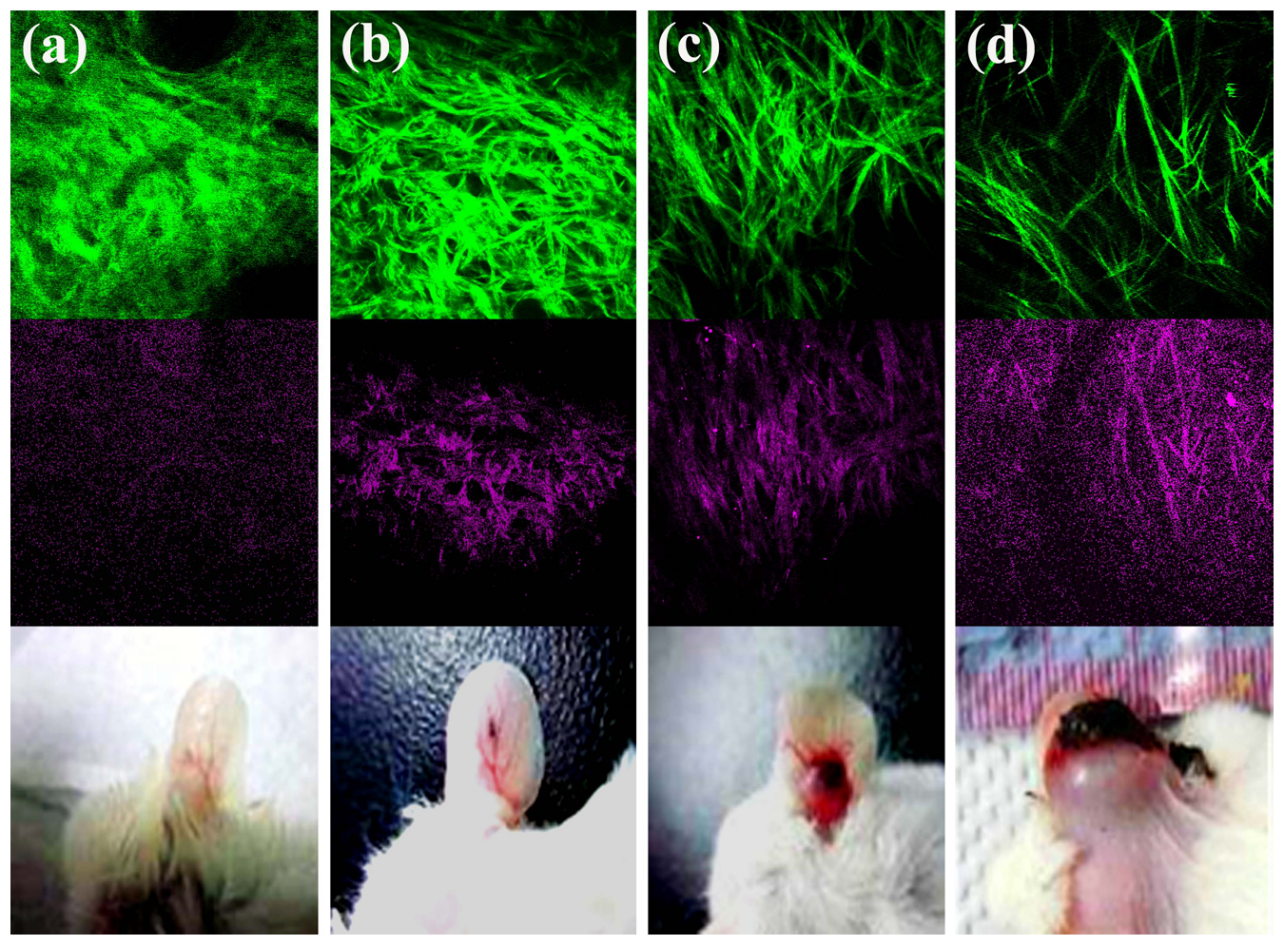
*In vivo* SHG (green) and THG (magenta) imaging of the collagen remodeling and the appearance of the mouse ear (a) before melanoma implantation and (b) 2 weeks, (c) 3 weeks, and (d) 4 weeks post melanoma implantation. (a) serve as the control group.

At a few days after the implantation of the melanoma cells, we can easily recognize strong TPF and THG contrasts *in vivo*, which were not present before implantation (Fig. 5). The co-localized TPF and THG signals reveal the distribution of the melanin and thus the location of the melanoma cells. Following this intrinsic feature, we can track the position of the melanoma at different stages without a label. At seven days after the implantation, the size of the tumor was so large that it can be observed with plain eyesight (Fig. 6, pictures). The black nodule grew from a 0.5 cm spot during the second week and finally reached a size of 2.0 cm. In the later stages of the melanoma development, invasion and collagen remodeling in the regions of the sebaceous glands, could be clearly observed with the SHG modality (Fig. 6). All of the vessel networks disappeared, and the “cable-like” collagens appeared with a high directionality (Fig. 6d).

### Quantitative analyses on the baseline variations of collagens in normal mice ears

It is well known that collagen structures are affected by age, sex, and body region^57^. To determine whether the structural changes of the collagen fibers adjacent to tumors are significant enough to serve as diagnostic indices, we first examined the baseline variations of the collagen structures in three mice (A, B, and C). For each mouse, we analyzed baseline variations due to tissue regions and depth. Based on these baseline values, we investigated whether the injection itself will cause the change of collagen structures by injected fluids or induced inflammation. To ensure this, we injected hyaluronic acid (HA) into the mouse ear to mimic the tumor implantation and observed the collagen networks in the same mouse and region for four consecutive weeks.

**Figure 7:**
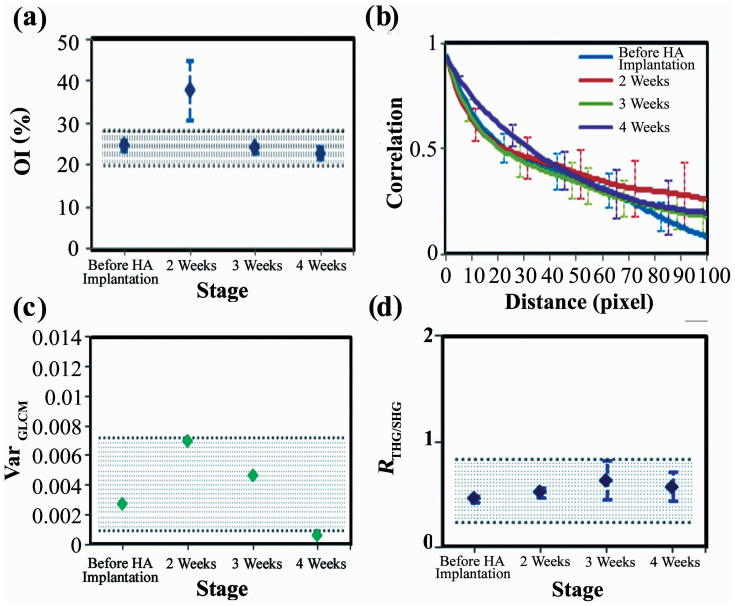
The (a) OI, (b) GLCM trace, (c) *Var_GLCM_*, and (d) *R*_THG/SHG_ of harmonic generation images after subcutaneous injection of HA. The areas with dense blue lines indicate the range of baseline values. The bars indicate the variation among different depth.

In the control study, for each normal mouse, we acquired SHG images over six different zones (zone1-6), in which we obtained 10 layers (0–18 μm) of imaging stacks at three different locations. In the quantitative analysis of the SHG and THG images, we avoided sebaceous glands (Fig. 3), vessel cavities (Fig. 3), and the bulky tumor regions where collagens were absent. Among the different normal mice, the overall average OI values were approximately 38% (Fig. S2a, *p* > 0.05). However, the standard deviations for the OIs in each mouse were large. This large deviation was caused by the large regional variation of the OI, which ranged from 20% to 45% (Fig. S2b). We found that zones 5 and 6 had significantly lower OI values than the other zones (*p* < 0.05). Even within the same zone, some of the standard deviations for particular zones were still large. From the skin surface down to 18 μm deep, there is no significant depth variation in the OI value (Fig. S2c). These results indicate that regional variation predominately produces the baseline variation of the OI values in the normal control mice. The relative variation of the means among different zones was approximately ±16%. To represent the range of the baseline values, we plotted an area with dense blue lines in the analysis (Fig. 7a).

Regarding GLCM analyses, there were no significant individual differences in the GLCM traces (Fig. S3a) among normal mice. In the different zones, significant changes in the GLCM values were observed (Fig. S3b). In the regional variation analysis of the GLCM values, we omitted zone 2 because this zone is not easily flattened. The correlation curve for zone 6 decreased much more quickly than the other five zones as the pixel distance increased. Moreover, the correlation of zone 6 dropped to 50% lower than zone 3 with increased pixel distances. The GLCM traces did not change greatly at different depths or after subcutaneous injection of HA (Fig. S3c and Fig. 7b). Except for the decay of correlation, we further developed a new index *Var_GLCM_* to evaluate the fluctuation of GLCM traces. To calculate this index, over all pixel distances, we summed the square of correlation fluctuation relative to the slowly varying background. Similar to the results of GLCM, it shows a large (±167%) regional variation (Fig. S4) and HA injection didn't deviate the value away from the range of baseline ones (Fig. 7c).

Because OI, GLCM, and *Var_GLCM_* all showed predominantly regional variation, we focused on the zone and depth analyses with the *R*_THG/SHG_. In the analysis of different zone, we omitted the depth from 0 μm to 2 μm due to the strong THG signals contributed by the epithelial cells at the surface of the skin. At each zone, we calculate *R*_THG/SHG_ at different depth. The *R*_THG/SHG_ exhibited regional variation ranging from 0.29 (zone 1) to 1.06 (zone 2) (Fig. S5a). The relative variation of the means among different zones was approximately ±86%. In the depth variation study, at each depth, we chose 50 locations to evaluate the mean and variation of *R*_THG/SHG_ values. In contrast to the OI and GLCM values, the *R*_THG/SHG_ showed a decay with increased depth (Fig. S5b). This might be due to the fact that THG signals decay faster than SHG signals when the spot size was enlarged at deep imaging depth. For the HA injection test, at each time point, we averaged the results of different depth in the same zone. Following up at the same location, there were no significant differences on the *R*_THG/SHG_ within 4 weeks (Fig. 7d). All of the values were within the baseline values, which is the range of the mean measured in Fig. S5a.

These control experiments validate that collagen structures have large regional variation. An HA injection won't cause significant changes of quantitative results except for a short-term change on OI values.

### Characteristic features of collagen remodeling after melanoma implantation

**Figure 8:**
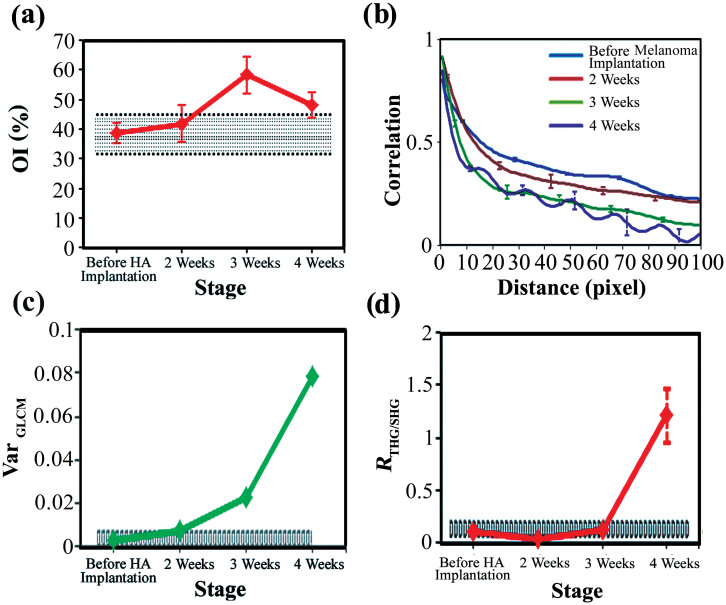
The (a) OI values, (b) GLCM traces, (c) VarGLCM values, and (d) RTHG/SHG values of the collagen networks revealed by the in vivo SHG images of mice before the melanoma implantation and at 2 weeks, 3 weeks, and 4 weeks post melanoma implantation. The areas with dense blue lines indicate the ranges of the baseline variation. The bars indicate the variation among different depths.

Understanding the range of baseline values for each quantitation method, we then tracked the tumor associated collagen at the fix position of the same mouse for four weeks. Before the implantation of the melanoma cells, the collagen OI was approximately 38%. At the 3 weeks post melanoma implantation, the OI increased to approximately 58% (Fig. 8a). This difference is significantly higher than the baseline variation (area with dense blue lines in Fig. 8a). In the GLCM analyses, we found that the short-distance correlation of the collagen textures declined faster as the tumor grew (Fig. 8b). At 4 weeks post-implantation, the correlation fell below 0.5 at a distance 30% shorter than that before the melanoma implantation. Another tumor-related feature is the oscillation of GLCM traces. To quantitatively analyze the oscillation of GLCM traces, we removed the slow varying background of GLCM traces (Fig. S6) and calculated the corresponding *Var_GLCM_*. The value was increased quickly from 0.015 to 0.078, which was quite sensitive to the tumor growth. The *R*_THG/SHG_ started with a low ratio of 0.03 at 2 weeks and then increased to 1.21 at 4 weeks post-implantation. The value at the fourth week was obviously higher than the baseline values.

**Figure 9:**
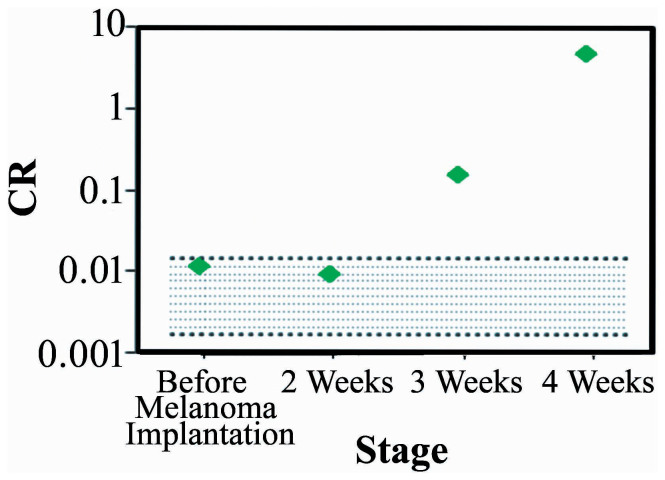
The result of CR obtained from mice before implantation and 2 weeks, 3 weeks, and 4 weeks post melanoma implantation.

Finally we multiplied the OI, *Var_GLCM_*, and *R*_THG/SHG_ values together to generate an integrated index of collagen remodeling (CR). We found it started from a small value of 0.01 at 2 weeks and then raised to 4.58 at 4 weeks post-implantation (Fig. 9). The overall change is about 400-times larger than those in 0–2 weeks. The CR value at 3 weeks post-implantation was already higher than the range of baseline values, which is calculated from the multiplication of baseline variation measured in Fig. 8a, 8c, and 8d.

## Discussion

Through quantitative analyses of the SHG and THG *in vivo* images, we found that the collagen structures have an obvious regional variation in the normal control mice. Furthermore, the *R*_THG/SHG_ index has depth dependency. Therefore, to reduce the baseline variation and extract the diagnostic features of the tumor-induced collagen remodeling, it is necessary to acquire sectioning images at the same locations and at a fixed depth. After implanting HA in zone 5, there was a significant increase in the OI at the same location in the first 2 weeks, which may have been caused by inflammation. After 3 weeks, the OI values returned to the original levels (Fig. 7a). After implanting melanoma, the GLCM trend agrees with a previous investigation on pancreatic tumor xenografts^48^ but not with a study on excised human ectocervical tissues^45^. These opposite trends may arise from the different types of tumors studied or the fact that the human specimen study was not a time-course or self-referenced comparison at the same sites. Moreover, a periodic fluctuation appeared in the GLCM trace at 3 weeks post-implantation, and this fluctuation became large and clear at 4 weeks post-implantation. We also found that collagens with greater directivity have fluctuations in the GLCM traces (Fig. 8a, Fig. 8b). Analyzing this unique fluctuation around the slowly varying GLCM traces, we define a new quantitative index *Var_GLCM_* and found it quite sensitive to the growth of melanoma (Fig. 8c). The decrease of *R*_THG/SHG_ in its early stage was due to an increase in the intensity of the SHG signal (Fig. 6b, green), which might be caused by fibrosis around the tumor^58^. Then, the intensities of the THG images increased as the tumor grew (Fig. 6d, magenta), indicating an inhomogeneous melanoma microenvironment. Consequently, the *R*_THG/SHG_ value increased drastically to 1.21 at 4 weeks post-implantation. Using integrated index of CR, finally, we got a more sensitive measure on the collagen remodeling of melanoma. At 4 weeks post-implantation, the value was raised 400-times higher than that before melanoma implantation.

Through these quantitative analyses of the *in vivo* SHG and THG images, we successfully extracted the diagnostic features of collagen remodeling in a melanoma microenvironment. By monitoring collagen adjacent to the cancer at the same locations, we reduced the effects of baseline variation and found that the melanoma-remodeled collagens have quantitative image features of a higher OI, a short-range texture correlation, periodic fluctuation in the GLCM traces, higher *Var_GLCM_* and *R*_THG/SHG_ at 4 weeks post-implantation. These indices together provide new dimensions for the diagnosis of melanoma. As a result, this least invasive HGM virtual optical biopsy has the potential for application to the diagnosis and screening of melanoma in a clinical setting. In the future, we will perform a human clinical trial to determine whether melanoma patients have similar collagen features around tumor sites.

## Methods

### Laser source and nonlinear optical microscope

Our laboratory-built nonlinear optical microscopy system was excited with a 1,250 nm femtosecond Cr:forsterite laser pumped by a Yb-doped fiber laser. The average mode-locked output power was approximately 500 mW with a 110 MHz pulse repetition rate. Our laser scanning microscope employed a commercial confocal scanning system (FV300, Olympus, Tokyo, Japan) combined with an upright microscope (BX51, Olympus, Tokyo, Japan). The collimated laser beam was scanned via dual-axis high-speed galvanometer mirrors and focused with an 60× infrared water-immersion objective with 1.2 numerical aperture (UPLANSAPO, Olympus, Tokyo, Japan). The power at the sample was 90 mW and the laser is stable. The nonlinear optical signals generated, such as the TPF, SHG, and THG signals, were epi-collected with the same objective. The SHG and THG signals were reflected by the multiphoton dichroic beam splitter (FF665-DI02, Semrock, Rochester, NY) and then separated by the 490 nm-edged dichroic beam splitter (490DRXR, Chroma technology, Bellows Falls, VT) into two individual photomultiplier tubes (PMTs; R4220P for THG and R943-02 for SHG, Hamamatsu, Bridgewater, NJ). The TPF signals passed through the multiphoton dichroic beam splitter and were back-propagated to a built-in PMT in the FV300 scanner. Appropriate band-pass filters were placed before the SHG (HQ615/30X, Chroma technology, Bellows Falls, VT) and THG (D410/30X, Chroma technology, Bellows Falls, VT) PMTs to raise the signal-to-noise ratio, and a color filter (CG-KG-5, CVI, Albuquerque, NM) was placed before the 490DRXR to block the residual photons of the excitation laser. The signals detected by the PMTs were amplified and sampled with an FV300 system, resulting in 512 × 512-pixel images at a frame rate of 3 Hz.

### Tumor cell line

The B16-F10 melanoma cell line was used for the tumor model in this experiment. The source of the B16-F10 cells (Strain Ethnicity: C57BL/6J) was the Food Industry Research and Development Institute, which originally obtained the cell line from the American Type Culture Collection (ATCC; number: CRL-6322). The culture medium used was 90% Dulbecco's modified Eagle's medium with 4 mM L-glutamine adjusted to contain 1.5 g/L sodium bicarbonate and 4.5 g/L glucose plus 13% fetal bovine serum (FBS). The melanoma cells were cultured in a humidified atmosphere of 5% CO_2_ at 37°C and were harvested every three days with 4 mM ethylene glycol tetraacetic acid to avoid the membrane-altering effects of trypsin.

### Animal model and anesthesia

All the protocols of animal experiments were approved by Institutional Animal Care and Use Committee of National Taiwan University Hospital (IACUC-NTUH). The melanoma cells were implanted into C57BL/6-C2J mice, which were purchased from the Animal Center of National Taiwan University Hospital. The melanoma cells were suspended in phosphate-buffered saline and subcutaneously implanted into the ears of 4 weeks old male C57BL/6-C2J mice. The injection amounts were 1.5 × 10^6^ melanoma cells in a volume of 30 μL. All of the mice were raised in acrylic cages. The body weights were approximately 20–25 g at the beginning of the experiment. All of the mice were monitored twice a week for tumor development and were sacrificed when the dimensions of the swelling tumor reached 8 cm^3^ or the mice exhibited cachexia in accordance with the guidelines of the IACUC-NTUH. Mild swelling and redness of the mouse ear was noted at 4–5 days after the implantation of the melanoma cells. An obvious nodule was noted approximately one week after the melanoma-cell implantation. We took images using nonlinear optical microscopy of the same mouse ear at least twice a week for 3 weeks.

Regarding anesthesia, isoflurane was used in our experiment for its effectiveness, lack of side-effects, and rapid wash-out in continuous time-course imaging and prolonged experimental observations. We continuously observed the reflexes and vital signs (94–163 breaths/min, 325–780 beats/min, 37.5°C) of the anesthetized mice. We concurrently maintained the body temperature with a small warm bag during the entire period the mice were under anesthesia until recovery.

### Orientation index (OI) analysis

The OI quantitative parameter has been widely used to analyze the directionality of a collagen fiber and is calculated as follows^34^:
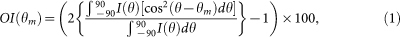
where *I(θ)* is the spatial frequency-averaged intensity as a function of the angle *θ*^34^. We define the angle at which the *I(θ)* reaches its maximum as the dominant direction *θ_m_*. A 100% OI represents an image in which all of the fibers are aligned along the same direction, while a 0% OI represents a random orientation.

### Gray level co-occurrence matrix (GLCM) analysis

The gray level co-occurrence matrix (GLCM) analyzes the common occurrences of a gray-level value for all of the pairs of pixels in textured images^47^. There are two important parameters in the calculation process: the distance *d* between the two pixels and the orientation angle *θ* of the line connecting these two pixels. Using functions in MATLAB, we calculated the GLCM at a specific distance and orientation. Then, we normalized the GLCM according to
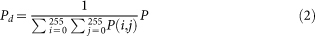
and calculated the correlation C of the GLCM according to the following equation:
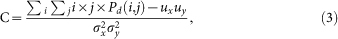
where *u_x_*, *u_y_*, *σ_x_*, and *σ_y_* are given by
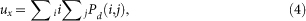

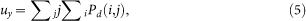

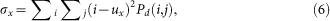

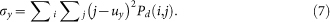
The values *u_x_*, *u_y_* and *σ_x_*, *σ_y_* are the means *u* and standard deviations *σ* of column *x* and line y of the matrix, respectively. If the correlation is high, this value will be close to 1. Then we plotted the traces of correlation versus the distance C(*d*) to evaluate the range of the texture correlation in the collagen images.

In order to quantitatively evaluate the feature of oscillation in GLCM traces of melanoma adjacent collagen, we first made adjacent average operation on C(*d*) traces to obtain the slowly varying background C_a_(*d*). Then we removed the background from original traces and obtain the variance:
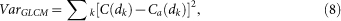
by the sum of squared difference between *C(d)* and *C_a_(d)* at each distance *d_k_*.

### Analysis on the ratio of the THG to the SHG

Dividing THG signals by SHG signals at each pixel, we calculated the ratio of *R*_THG/SHG_. We analyzed only the collagen networks and avoided the regions with sebaceous glands, cells, and vessels, where the SHG signal was absent. We chose 50 different regions for analysis in each image and averaged these regions to obtain the representative *R*_THG/SHG_ at that sectioning plane.

### Statistical analyses

All of the data are expressed as the means ± standard deviation in the figures. A one-way ANOVA, two-way ANOVA, linear regression analysis, and Student's unpaired *t*-test were performed using SPSS 20.0 for Windows (IBM Software). A value of *p* < 0.05 was considered statistically significant.

## Author Contributions

The experiments were designed and conducted by T.M., P.C. and T.Y. cultured cancer cells, handled the animals, made tumor implantation, acquired images, and performed quantitative analysis. Z.U. helped the cell culture.

## Supplementary Material

Supplementary Information Supplementary information
