# Supplementary material for: In vivo Quantification of the Structural Changes of Collagens in a Melanoma Microenvironment with Second and Third Harmonic Generation Microscopy
Source: Sci Rep. 2015 Mar 9;5:8879. doi: 10.1038/srep08879 (PMC4352861; doi:10.1038/srep08879)
Supplement: Supplementary Information [file srep08879-s1.pdf]

Supplementary Information

In vivo Quantification of the Structural Changes of Collagens in a Melanoma Microenvironment with Second and Third Harmonic Generation Microscopy

Pei-Chun Wu[†], Tsung-Yuan Hsieh[†], Zen-Uong Tsai[‡], and Tzu-Ming Liu[†‡]*

[†]Institute of Biomedical Engineering, National Taiwan University, Taipei 10617, Taiwan
[‡]Molecular Imaging Center, National Taiwan University, Taipei 10617, Taiwan

Zones of observation and reference marks of orientation on the mice ear pinnae. To study the regional variation of the collagen structures revealed by the *in vivo* SHG and THG microscopy, we partitioned the ear pinna of a mouse into nine observation zones (Fig. S1). The dimension of each zone was approximately 3 × 3 mm. Before the implantation of the melanoma cells, the SHG and THG sectioning images of the collagen networks in each zone were acquired as a normal control. After the implantation, for the convenience of positioning in the follow-up observations, we cut a triangular piece from the mouse ear pinna at zone 8 (Fig. S1, along the yellow lines). The apex of the sharp notch served as a positional origin for locating the tumor microenvironment in each follow-up observation.

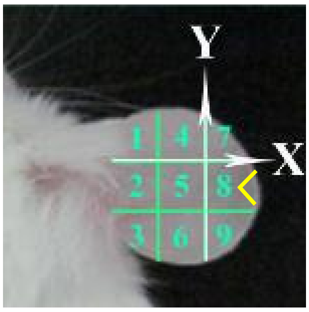

Figure S1. Partition of the observation zones on the mouse ear pinna. We marked the mouse ear by cutting a triangular notch (approximately 0.5 mm long on each side) for the purpose of positioning in the follow-up observations.

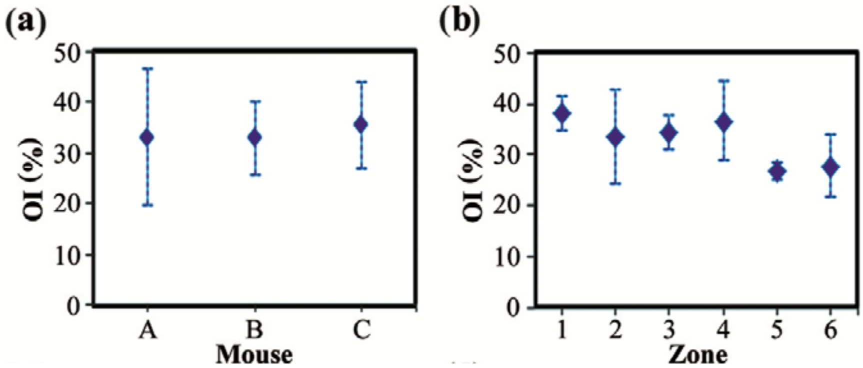

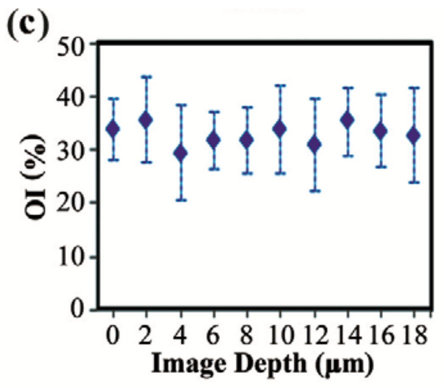

Figure S2. The (*a*) individual, (*b*) regional, and (*c*) depth variation of the OI in normal mice. The bars indicate the standard deviations among (*a*) different locations and zones and (*b*) (*c*) different locations.

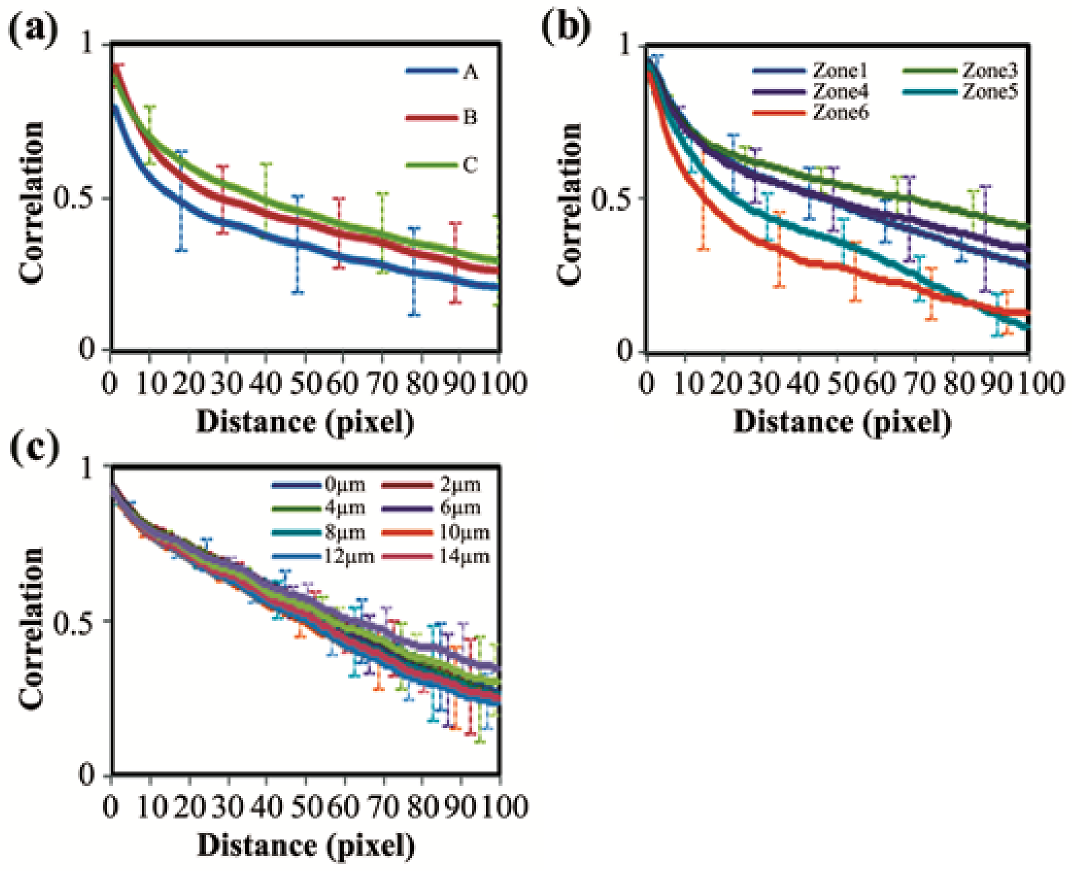

Figure S3. The (*a*) individual, (*b*) regional, and (*c*) depth variation of GLCM values in normal mice. The bars indicate the standard deviations among (*a*) different locations and zones, (*b*)(*c*) different locations, and (*d*) different depths.

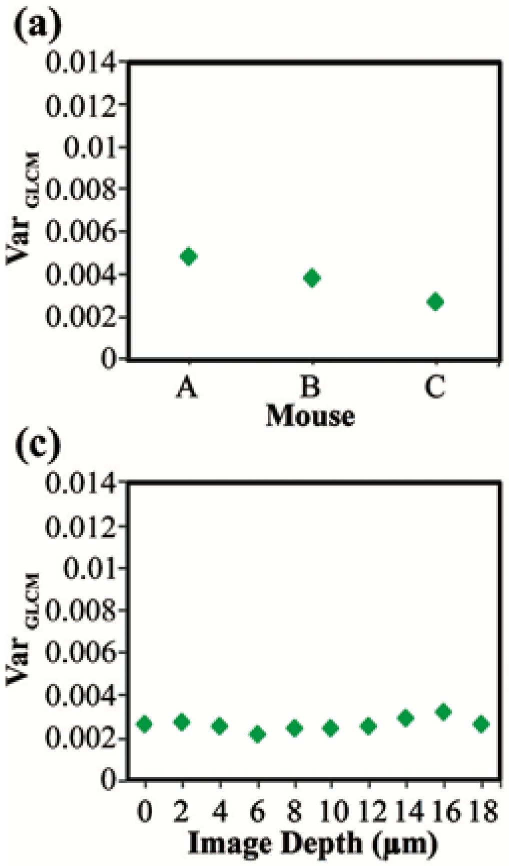

Figure S4. The (*a*) individual, (*b*) regional, and (*c*) depth variation of Var_{GLCM} values in normal mice.

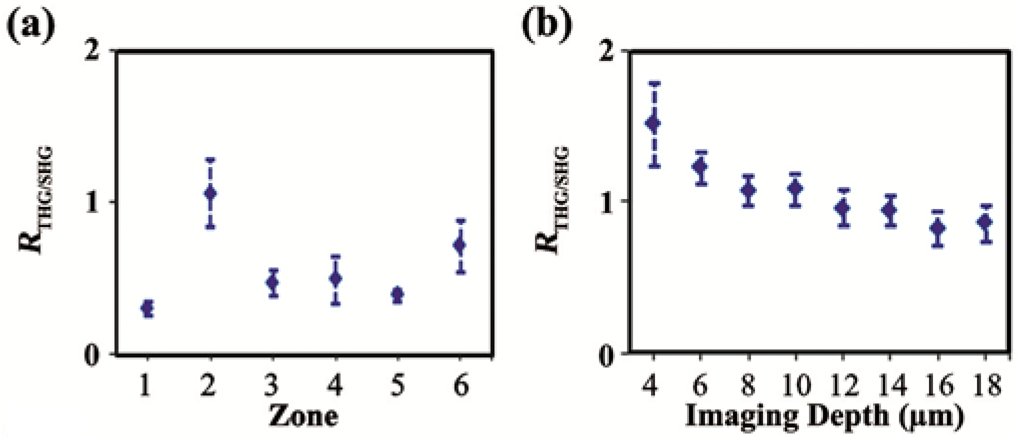

Figure S5. The (*a*) regional and (*b*) depth variation of the $R_{THG/SHG}$. The bars indicate the standard deviations among (*a*) different depth and (*b*) different locations.

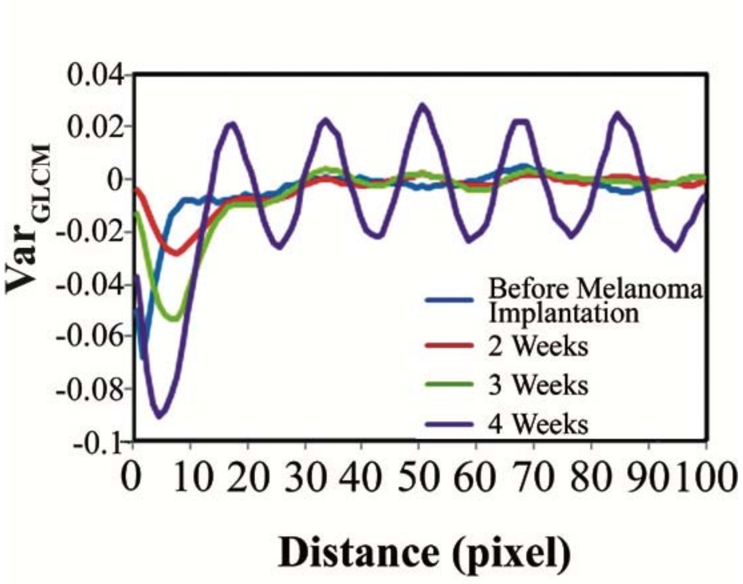

Figure S6. The fluctuation of the texture correlation relative to the slowly varying background of GLCM traces.
